# Supplementary material for: ‘You’re still the same person, but you’re just seeing a bit less’: a qualitative study of the impact of vision impairment on mental well-being in children and young people with inherited eye disease in England
Source: BMJ Open. 2026 Sep 28;16(9):e125977. doi: 10.1136/bmjopen-2026-125977 (PMC13629961; doi:10.1136/bmjopen-2026-125977)
Supplement: Supplementary file 1 [file bmjopen-2026-125977.reviewer_comments.pdf]

PEER REVIEW HISTORY

BMJ Open publishes all reviews undertaken for accepted manuscripts. Reviewers are asked to complete a checklist review form and are provided with free text boxes to elaborate on their assessment. These free text comments are reproduced below.

ARTICLE DETAILS

Title (Provisional)

'You're still the same person, but you're just seeing a bit less': A qualitative study of the impact of vision impairment on mental wellbeing in children and young people with inherited eye disease in England

Authors

Canavan, Rachael F; Tibber, Marc Samuel; Tailor, Ethan R; Dekker, Tessa M; Michaelides, Michael; Oluonye, Ngozi; Sumodhee, Dayyanah; Crossland, Michael D.

VERSION 1 - REVIEW

**Reviewer**	**1**
**Name**	**Wittich, Walter**
**Affiliation**	**Universite de Montreal, School of Optometry**
**Date**	**16-Aug-2026**
**COI**	**None**

Thank you for giving me the opportunity to review this interesting manuscript. The comments below are intended to further elevate the already excellent work the authors present.

The overall purpose of the study is clear: to explore mental wellbeing among adolescents with vision impairment caused by inherited macular disease and identify factors that support or undermine wellbeing. The introduction ultimately states that the study sought insight into both the impact of VI on wellbeing and participants' views regarding optimal support and interventions. However, the objective needs greater precision. The manuscript alternates among "impact," "association," "mechanisms," and "factors that enhance and limit wellbeing." In a qualitative study, particularly an RTA study, "explore experiences and perceived influences" would be preferable to language implying that causal mechanisms or associations are being established.

There is also an inconsistency: the introduction describes adolescents as "aged 10–20 years", whereas eligibility was "13–18 years". This should be harmonized.

I would ask the authors to state one explicit research objective at the end of the Introduction and use that formulation consistently throughout.

The abstract captures the study design and the four themes well. The stated four themes correspond closely to those presented in the Findings. Several details should nevertheless be addressed. First, the abstract identifies the setting as low-vision/genetic eye disease clinics in a tertiary hospital, whereas the Methods state that participants were recruited from “both a hospital low vision clinic and a UK vision-impairment charity”. Second, referring generically to “children and young people” obscures that the directly interviewed young participants numbered only six and were 13–18 years old. Please include the sample size in the “Participants” section. Third, the conclusion uses relatively causal language like “factors linking VI to reduced mental wellbeing” which is stronger than warranted by qualitative participant accounts.

Methods: Semi-structured qualitative interviewing is very appropriate for exploring the lived experience of wellbeing, adaptation, identity, support, stigma, participation and future aspirations among adolescents with inherited vision impairment.

The decision to include both adolescents and parents is potentially a strength because it provides different vantage points. However, the authors should be clearer about what kind of dataset this choice creates. There are six young-person participants but twelve parent participants, and it is not clear that the two groups constitute matched parent-child dyads. Therefore, parent perspectives should not be treated as confirmation or “validation” of adolescent accounts. The manuscript currently describes interviewing parents as providing “additional validation of the themes identified.” I think that wording is conceptually problematic, particularly within RTA. These are different situated perspectives, which may converge or diverge; the divergences themselves are analytically interesting.

One of the manuscript's more interesting observations is that parents sometimes interpreted friendship and family consequences more negatively than the young people themselves. That discrepancy deserves more analytic attention rather than being framed primarily through triangulation or validation.

There is considerable methodological detail: recruitment criteria, purposive sampling, interview platform, setting, interview guide development, recording, transcription, NVivo version, six analytic phases, critical-realist positioning, reflexivity and PPI are all described.

My principal concern is the description of RTA. The manuscript states that two researchers worked “independently on a line-by-line basis” and simultaneously positions the analysis as Braun and Clarke-style reflexive thematic analysis. The authors need to clarify what “independently” means and how the two researchers' analyses were subsequently brought together. Were codes compared? Was agreement sought? Were themes developed collaboratively? Were differences deliberately retained as reflexive resources?

This matters because RTA does not normally conceptualise multiple independent coders as a mechanism for establishing coding reliability or objective agreement. Researcher subjectivity is understood as part of knowledge production rather than as error that should be eliminated. The manuscript cites Braun and Clarke's quality guidance, making this internal methodological coherence particularly important. The authors should therefore give a fuller account of the analytic process after initial coding and explain how their approach is consistent with their stated RTA methodology.

Ethical approval is clearly reported, including the HRA/REC details, and written informed consent and video-recording consent are stated. However, because participants were aged 13–18, I would expect more detail about consent/assent for minors and parental/guardian consent, including how capacity to consent was handled for younger participants.

More importantly, some interviews elicited accounts of self-harm and suicidal ideation. Given that this was a mental-wellbeing study involving minors, the manuscript should describe any distress, safeguarding and risk-management protocol used during interviews. This might include procedures for responding to disclosure of current suicidal thoughts, self-harm or serious psychological distress and circumstances under which confidentiality would be overridden. The absence of this information is notable for this population and topic.

“Mental wellbeing” itself is never particularly well defined conceptually. The manuscript sometimes uses wellbeing, mental wellbeing, mental health, psychological wellbeing and emotional wellbeing almost interchangeably. The interview guide likewise moves between “mental health and well-being.” A brief conceptual definition would help readers understand what phenomena the authors intended to explore and would make the interpretation of themes such as independence, participation, stigma and family support more coherent.

One cited study by the authors is “under review,” which limits readers' ability to evaluate the comparison being made.

Ecological Systems Theory appears quite prominently only in the Discussion rather than clearly framing the study prospectively. If it is intended as a post hoc interpretive lens, the authors should state this explicitly. If it informed analysis, it should be introduced in the Methods.

The Findings section is one of the strongest parts of the manuscript. There is extensive use of vivid participant quotations, and the quotations frequently demonstrate complexity rather than presenting only one uniform experience. The Findings could nevertheless be tightened considerably. At more than 8,000 words overall, there are many quotations conveying similar points. Reducing repetition would sharpen the analytic contribution.

I would also encourage the authors to distinguish more explicitly between CYP-derived findings and parent interpretations. With twice as many parent participants, there is a risk that the thematic narrative unintentionally becomes predominantly a parental account of adolescent wellbeing. Some interpretive statements also go beyond the data. For example,

where differences between parent and CYP reports are suggested to indicate that parents may be “adept at masking challenges,” this is plausible but not the only explanation. Such passages should either be framed explicitly as interpretation or moved into the Discussion.

The core interpretation that wellbeing is shaped by emotional, social, functional and systemic influences is well supported. The Discussion appropriately identifies acceptance, adaptation, self-efficacy, stigma/discrimination, social connection and counselling as important perceived influences. However, the transition from qualitative findings to service recommendations occasionally becomes too prescriptive. For example, statements that particular services “should be provided”, including age-appropriate Eye Clinic Liaison Officers, counselling and peer support, imply intervention effectiveness that this study did not test. Instead, I suggest language such as “participants' accounts support consideration of…” or “these findings identify potentially important targets for intervention that warrant evaluation.”

The ecological interpretation is potentially valuable, but it currently feels somewhat appended to the findings. A more explicit mapping of the four themes across individual, interpersonal, institutional and societal levels could turn this into a stronger conceptual contribution.

The authors acknowledge the restricted diagnosis, tertiary specialist setting, possible selection bias, limited generalisability and small sample. These are appropriate. I would add at least four limitations: only six adolescents were interviewed compared with twelve parents; perspectives appear not necessarily to represent matched adolescent-parent dyads; requiring sufficient English for an online interview excluded participants requiring interpreting or other language support, potentially reducing socioeconomic/cultural inclusiveness; all young people were in mainstream education and without learning disability, further restricting transferability.

I would also reconsider the statement that the authors “feel that a level of saturation was achieved.” Saturation is contested generally and particularly sits somewhat awkwardly with reflexive TA, where themes are actively generated rather than assumed to become objectively exhausted. Given the authors' own methodological framework, they could instead justify the sample in terms of information richness, study scope and the focused nature of the participant group.

VERSION 1 - AUTHOR RESPONSE

Reviewer: 1
Prof. Walter Wittich, Universite de Montreal
We thank Professor Wittich for his kind and constructive review.

Comments to the Author:
Thank you for giving me the opportunity to review this interesting manuscript. The

comments below are intended to further elevate the already excellent work the authors present.

The overall purpose of the study is clear: to explore mental wellbeing among adolescents with vision impairment caused by inherited macular disease and identify factors that support or undermine wellbeing. The introduction ultimately states that the study sought insight into both the impact of VI on wellbeing and participants' views regarding optimal support and interventions. However, the objective needs greater precision.

We have tightened the objective to: 'To explore the influence of VI on mental wellbeing in children and young people (CYP) with inherited macular disease and experiences perceived to enhance and limit their wellbeing' in the abstract (lines 31-33) and method (line 121-123).

The manuscript alternates among "impact," "association," "mechanisms," and "factors that enhance and limit wellbeing." In a qualitative study, particularly an RTA study, "explore experiences and perceived influences" would be preferable to language implying that causal mechanisms or associations are being established.

We have reviewed this thoroughly and have removed terms which imply a causal relationship.

There is also an inconsistency: the introduction describes adolescents as "aged 10–20 years", whereas eligibility was "13–18 years". This should be harmonized.

As our inclusion criterion (age 13-18 years) was narrower than the World Health Organisation definition of adolescence (10-19 years), we have replaced 'adolescents' with 'Children and Young People' or CYP throughout, including in the title.

I would ask the authors to state one explicit research objective at the end of the Introduction and use that formulation consistently throughout.
This has been added as requested (lines 115-116).

The abstract captures the study design and the four themes well. The stated four themes correspond closely to those presented in the Findings. Several details should nevertheless be addressed. First, the abstract identifies the setting as low-vision/genetic eye disease clinics in a tertiary hospital, whereas the Methods state that participants were recruited from "both a hospital low vision clinic and a UK vision-impairment charity".

The abstract has been corrected to read 'Low vision and ophthalmology clinics in England'. All participants were under low vision and ophthalmology care (line 38).

Second, referring generically to "children and young people" obscures that the directly interviewed young participants numbered only six and were 13–18 years old. Please include the sample size in the "Participants" section.

We refer to ‘children and young people’ as, although our adolescent participants were aged 13-18, the parents were asked to reflect on their child’s wellbeing from diagnosis (which may have been before the age of 13) onwards. We have included the sample size in the ‘participants’ section as requested (line 39-41).

Third, the conclusion uses relatively causal language like “factors linking VI to reduced mental wellbeing” which is stronger than warranted by qualitative participant accounts.

This has been changed to ‘Experiences perceived to influence mental well-being’ (line 54).

Methods: Semi-structured qualitative interviewing is very appropriate for exploring the lived experience of wellbeing, adaptation, identity, support, stigma, participation and future aspirations among adolescents with inherited vision impairment.

The decision to include both adolescents and parents is potentially a strength because it provides different vantage points. However, the authors should be clearer about what kind of dataset this choice creates. There are six young-person participants but twelve parent participants, and it is not clear that the two groups constitute matched parent-child dyads. Therefore, parent perspectives should not be treated as confirmation or “validation” of adolescent accounts. The manuscript currently describes interviewing parents as providing “additional validation of the themes identified.” I think that wording is conceptually problematic, particularly within RTA. These are different situated perspectives, which may converge or diverge; the divergences themselves are analytically interesting.

We have clarified that not all of the parent participants had a child who participated in the study (and not all of the CYP participants had a parent who participated) in the method (line 140-142) and discussion (line 800-802). We have not reported which participants were part of a parent:child dyad to preserve anonymity. We have removed the reference to ‘validation’ and instead highlighted areas where perspectives converged and diverged (lines 802-809).

One of the manuscript's more interesting observations is that parents sometimes interpreted friendship and family consequences more negatively than the young people themselves. That discrepancy deserves more analytic attention rather than being framed primarily through triangulation or validation.

We have delved into this further in the discussion (lines 799-809). As well as speculating that this may represent masking by parents, we have added a brief discussion of the disability paradox, which may present an alternative explanation.

There is considerable methodological detail: recruitment criteria, purposive sampling, interview platform, setting, interview guide development, recording, transcription, NVivo version, six analytic phases, critical-realist positioning, reflexivity and PPI are all described. My principal concern is the description of RTA. The manuscript states

that two researchers worked “independently on a line-by-line basis” and simultaneously positions the analysis as Braun and Clarke-style reflexive thematic analysis. The authors need to clarify what “independently” means and how the two researchers' analyses were subsequently brought together. Were codes compared? Was agreement sought? Were themes developed collaboratively? Were differences deliberately retained as reflexive resources? This matters because RTA does not normally conceptualise multiple independent coders as a mechanism for establishing coding reliability or objective agreement. Researcher subjectivity is understood as part of knowledge production rather than as error that should be eliminated. The manuscript cites Braun and Clarke's quality guidance, making this internal methodological coherence particularly important. The authors should therefore give a fuller account of the analytic process after initial coding and explain how their approach is consistent with their stated RTA methodology.

This has been expanded (lines 176-184).

Ethical approval is clearly reported, including the HRA/REC details, and written informed consent and video-recording consent are stated. However, because participants were aged 13–18, I would expect more detail about consent/assent for minors and parental/guardian consent, including how capacity to consent was handled for younger participants.

We have clarified that participants aged 16 years or over provided written informed consent prior to taking part, and that participants under 16 provided verbal or written assent and written informed consent was obtained from a parent or carer (lines 224-227).

More importantly, some interviews elicited accounts of self-harm and suicidal ideation. Given that this was a mental-wellbeing study involving minors, the manuscript should describe any distress, safeguarding and risk-management protocol used during interviews. This might include procedures for responding to disclosure of current suicidal thoughts, self-harm or serious psychological distress and circumstances under which confidentiality would be overridden. The absence of this information is notable for this population and topic.

We confirm that we had a detailed suicide and self-harm risk protocol, which has been discussed (lines 165-167, supplemental material). An edited version, masking internal telephone numbers, has been provided as supplementary material.

“Mental wellbeing” itself is never particularly well defined conceptually. The manuscript sometimes uses wellbeing, mental wellbeing, mental health, psychological wellbeing and emotional wellbeing almost interchangeably. The interview guide likewise moves between “mental health and well-being.” A brief conceptual definition would help readers understand what phenomena the authors intended to explore and would make the interpretation of themes such as independence, participation, stigma and family support more coherent.

We used the World Health Organisation definition of mental health as being ‘a state of mental well-being that enables people to cope with the stresses of life,

realize their abilities, learn and work well, and contribute to their community.' Importantly, our study was not designed only to identify risks of mental ill-health conditions such as depression, anxiety or post-traumatic stress disorder, despite their increased prevalence in people with vision impairment (lines 124-128).

One cited study by the authors is "under review," which limits readers' ability to evaluate the comparison being made.

Sadly this is still under review (despite being submitted significantly earlier than the current manuscript!). We have added a reference to the abstract of a conference presentation so the reader can find more detail on this previous work (lines 154, 813).

Ecological Systems Theory appears quite prominently only in the Discussion rather than clearly framing the study prospectively. If it is intended as a post hoc interpretive lens, the authors should state this explicitly. If it informed analysis, it should be introduced in the Methods.

This was an a posteriori interpretative lens. We have clarified this in the discussion (line 787).

The Findings section is one of the strongest parts of the manuscript. There is extensive use of vivid participant quotations, and the quotations frequently demonstrate complexity rather than presenting only one uniform experience. The Findings could nevertheless be tightened considerably. At more than 8,000 words overall, there are many quotations conveying similar points. Reducing repetition would sharpen the analytic contribution. I would also encourage the authors to distinguish more explicitly between CYP-derived findings and parent interpretations. With twice as many parent participants, there is a risk that the thematic narrative unintentionally becomes predominantly a parental account of adolescent wellbeing.

We have reduced the duplicated quotations, preferentially including more from CYP and fewer from parents, which has reduced the length of the 'findings' section from 6536 to 5482 words.

Some interpretive statements also go beyond the data. For example, where differences between parent and CYP reports are suggested to indicate that parents may be "adept at masking challenges," this is plausible but not the only explanation. Such passages should either be framed explicitly as interpretation or moved into the Discussion.

We have edited the findings section carefully to present only results directly supported by participants' reports, and have moved our interpretations to the discussion section.

The core interpretation that wellbeing is shaped by emotional, social, functional and systemic influences is well supported. The Discussion appropriately identifies acceptance, adaptation, self-efficacy, stigma/discrimination, social connection and counselling as important perceived influences. However, the transition from

qualitative findings to service recommendations occasionally becomes too prescriptive. For example, statements that particular services “should be provided”, including age-appropriate Eye Clinic Liaison Officers, counselling and peer support, imply intervention effectiveness that this study did not test. Instead, I suggest language such as “participants' accounts support consideration of…” or “these findings identify potentially important targets for intervention that warrant evaluation.”

We have edited the discussion carefully to reduce the strength of our recommendations, as suggested.

The ecological interpretation is potentially valuable, but it currently feels somewhat appended to the findings. A more explicit mapping of the four themes across individual, interpersonal, institutional and societal levels could turn this into a stronger conceptual contribution.
We have made the link between our four themes and the ecological systems theory clearer in the first paragraph of the discussion (lines 783-790).

The authors acknowledge the restricted diagnosis, tertiary specialist setting, possible selection bias, limited generalisability and small sample. These are appropriate. I would add at least four limitations: only six adolescents were interviewed compared with twelve parents; perspectives appear not necessarily to represent matched adolescent-parent dyads; requiring sufficient English for an online interview excluded participants requiring interpreting or other language support, potentially reducing socioeconomic/cultural inclusiveness; all young people were in mainstream education and without learning disability, further restricting transferability.

We agree and have expanded the limitations section (lines 830-852).

I would also reconsider the statement that the authors “feel that a level of saturation was achieved.” Saturation is contested generally and particularly sits somewhat awkwardly with reflexive TA, where themes are actively generated rather than assumed to become objectively exhausted. Given the authors' own methodological framework, they could instead justify the sample in terms of information richness, study scope and the focused nature of the participant group.

We have removed references to saturation, as suggested, and instead refer to ‘the focused nature of the participant group allowing thematic sufficiency to be reached through the richness of the data’ (line 837).
