# Supplementary material for: ‘You’re still the same person, but you’re just seeing a bit less’: a qualitative study of the impact of vision impairment on mental well-being in children and young people with inherited eye disease in England
Source: BMJ Open. 2026 Sep 28;16(9):e125977. doi: 10.1136/bmjopen-2026-125977 (PMC13629961; doi:10.1136/bmjopen-2026-125977)
Supplement: Supplementary file 2 [file bmjopen-16-9-s001.docx]

**SUPPLEMENTARY MATERIAL: INTERVIEW TOPIC GUIDES**

*Interview topic guide for CYP. (VI=visual impairment).*

|  | Interview question |
| --- | --- |
| 1. | When you first found out about your VI, how did it make you feel? |
| 2. | How does having VI make you feel now? |
| 3. | Can you share how your VI has changed different parts of your life? |
| 4. | Taking care of your mental health and well-being is super important. Have you found any good ways to take care of them? |
| 5. | What support have you been given? |
| 6. | When did you receive this support? |
| 7. | Do you belong to any groups or clubs for young people with VI? |
| 8. | What gets in the way of receiving support? |
| 9. | When you first found out about your VI, what helpful information or resources did you wish you had? |
| 10. | If a young person with VI you know is having a tough time with their well-being and mental health, what kind of support do you think would help them? |
| 11. | Have you ever been given any support that didn’t really help you |
| 12. | Why didn’t this support help? |
| 13. | When do you think it would be most helpful for a young person with VI to be offered support? |
| 14. | Where do you think a young person with VI would feel most comfortable getting support? |

*Interview topic guide for parents/carers. (VI=visual impairment).*

|  | Interview question |
| --- | --- |
| 1. | How would you describe your child’s well-being when they were first told they had this eye condition? |
| 2. | How would you describe your child’s well-being at the current time? |
| 3. | How would you describe your well-being? |
| 4. | What support does your child currently receive? |
| 5. | Are they members of any groups of people with VI, or do they know anyone else with VI? |
| 6. | Are you a member of any VI groups? Do you know any other parents of a child with VI? |
| 7. | What else would have helped your child/ you/ your family that you haven’t had? |
| 8. | Is there any support you’ve had which wasn’t helpful? |
| 9. | What support do you think we should offer to young people with VI who are struggling with their mental health and well-being? |
| 10. | What are the biggest hurdles to receiving support? |
| 11. | Where is the best place to give this support? |
| 12. | What do you think makes your child’s mental health and well-being better? |
| 13. | What do you think makes your child’s mental health and well-being worse? |
| 14. | As a family what is the most helpful thing you do for your mental health and well-being? |
