# Supplementary material for: ‘You’re still the same person, but you’re just seeing a bit less’: a qualitative study of the impact of vision impairment on mental well-being in children and young people with inherited eye disease in England
Source: BMJ Open. 2026 Sep 28;16(9):e125977. doi: 10.1136/bmjopen-2026-125977 (PMC13629961; doi:10.1136/bmjopen-2026-125977)
Supplement: Supplementary file 3 [file bmjopen-16-9-s002.docx]

### Suicide and deliberate self-harm Risk Management Protocol

We will apply this protocol if suicidality or deliberate self-harm is reported at any stage of the interviews.

All adverse events will be recorded in the study log, to include recording of any clinical symptoms, as well as a simple, brief description of the event, including times/dates as appropriate. This will be passed on to the GP if necessary. During interviews the PhD student will always have access to a senior (duty) member of the research team if needed to seek telephone advice or to attend in person, as well as the contact number of a clinician (Clinical Psychologist) on the team.

All adverse events will be recorded and reported to the Research Ethics Committee (REC) as part of the annual reports.

**At the start of the telephone call, ask the participant where they are (e.g. at home/work) and confirm the address.**

**Suggested script:**

“You’ve indicated that you’re having thoughts of harming or killing yourself and it’s really important for us to make sure that you’re receiving the right kind of support, so I’m going to ask some more questions about those feelings, ok?”

**Thoughts**

“Could you describe to me what kinds of thoughts you’ve been having?”

**Ideas**

“Have had had any thoughts about how you might consider ending your life?”

**Plans/Preparations**

“Have you made any plans or preparations to make an attempt on your life?”

**Past Attempts**

“Have you ever attempted suicide in the past?”

**Deliberate self-harm**

“Do you self-harm, e.g. cut yourself?”

**Preventative/Protective Factors**

“Is there anything that his holding you back from making an attempt on your life at the moment? For example, are you able to think about reasons for living, or particular people who are very supportive to you?”

**Immediate Danger**

“Do you feel there is any immediate danger that you will harm yourself in a way that will endanger your life?”

| Thoughts | No | Yes to one or more | | No | Yes to one or more | | Yes or No |
| --- | --- | --- | --- | --- | --- | --- | --- |
| Ideas | No |  |  | No |  |  | Yes or No |
| Plans/Preparations | No |  |  | No |  |  | Yes or No |
| Previous Attempt | No |  |  | No |  |  | Yes or No |
| Current self-harm | No |  |  | No |  |  | Yes or No |
| Preventative | Yes | Yes | | No | No | | Yes or No |
| Immediate Danger | No | No | | No | No | | Yes |
|  | 🡪 | 🡪 | 🡪 | | 🡪 | 🡪 | |
|  | Recommend speaking to GP and direct to support sources | Write to GP and direct to support sources | | | Call GP and direct to support sources | Call 999 and provide participant’s address; inform clinical Co-I or senior member of research team immediately | |

Support sources:

General

Samaritans	08457 90 90 90	Any time

Saneline	0845 767 8000	6pm – 11pm, any day

Mind Infoline	0300 123 3393	9am – 5pm, Mon – Fri

Vision specific

Macular Society Counselling Service	0330 3030 111 (over 16’s only)

Moorfields Children’s Counselling	*** **** **** ext **** (answerphone)

Moorfields Adults’ Counselling	*** **** *** (answerphone)

RNIB Helpline	0303 123 9999 8am – 8pm, Mon – Fri

EMERGENCY help for Moorfields patients: Mental Health Services at East London Foundation Trust. *** **** **** bleep ***
